# Efficacy of extraoral suction devices in aerosol and splatter reduction during ultrasonic scaling: A laboratory investigation

**DOI:** 10.34172/joddd.2021.033

**Published:** 2021-08-25

**Authors:** Sivaporn Horsophonphong, Yada Chestsuttayangkul, Rudee Surarit, Wannee Lertsooksawat

**Affiliations:** ^1^Department of Pediatric Dentistry, Faculty of Dentistry, Mahidol University, Bangkok Thailand; ^2^Dental Department, Rajavithi Hospital, Bangkok Thailand; ^3^Department of Oral Biology, Faculty of Dentistry, Mahidol University, Bangkok Thailand; ^4^Department of Pharmacology, Faculty of Dentistry, Mahidol University, Bangkok Thailand

**Keywords:** Aerosol contamination, Dental procedure, Extraoral suction, Splatter, Ultrasonic scaling

## Abstract

**Background.** Ultrasonic scaling generates aerosols and splatters contaminated with microorganisms, increasing the risk of disease transmission in the dental office. The present study aimed to evaluate the effectiveness of extraoral suction (EOS) units in aerosol and splatter reduction during ultrasonic scaling.

**Methods.** Ultrasonic scaling was conducted on a dental manikin headset to simulate a scaling procedure. Water containing *Lactobacillus acidophilus* at a concentration of 10^7^ colony-forming units per milliliter and 1% fluorescein solution was used as the water supply of the scaler. The scaling procedure was conducted with a high-volume evacuator (HVE) or the combination of HVE and an EOS unit. de Man–Rogosa–Sharpe agar plates were placed at different distances surrounding the dental chair. Filter papers were placed at various positions surrounding the oral cavity and on areas of the body.

**Results.** Bioaerosols were detected at every sampling site and could travel as far as 150 cm from the oral cavity. The combination of HVE and EOS significantly reduced the total number of bacterial colonies in the air (*P* < 0.001). Dissemination of the stain was in the range of 20 cm from the oral cavity. The maximum contaminated surface area was at the 4 o’clock position from the oral cavity. The combination of EOS and HVE significantly reduced the contaminated area (*P* < 0.05). The stain was also found on the wrists, chest, abdomen, and lap of the operator and assistant. The lap was the most contaminated area of the body.

**Conclusion.** EOS was effective in reducing the bioaerosols and splatters generated during ultrasonic scaling.

## Introduction

Cross-transmission of disease in the dental clinic can occur by direct contact with oral secretions from patients, contact with contaminated dental instruments or environmental surfaces in the working area, or inhalation of bioaerosols generated during dental treatments. This creates a possible risk of infection in dental healthcare workers and patients.^1,2^ Many dental procedures generate aerosols and droplets contaminated with saliva, blood, dental plaque, and microorganisms from the oral cavity.^1,3^ The airborne particles produced by the dental treatment could cause the transmission of many infectious diseases, such as tuberculosis, colds, pneumonic plague, measles, influenza, and severe acute respiratory syndrome (SARS).^1,4^

The pandemic spread of the coronavirus disease 2019 (COVID-19) caused by the SARS-associated coronavirus-2,^5^ raises concerns about infection control and cross-transmission of the disease in the dental clinic. Since the disease is easily transmissible via an airborne route and most dental procedures generate aerosols and airborne droplets that contain microorganisms from the oral cavity,^1^ dental procedures could increase the risk of infection and transmission of the disease.

Ultrasonic scaling, a common treatment generally performed in the dental clinic, is considered to generate the most significant amount of aerosol contamination, increasing the risk of infection in dental practice.^1,6^ Because of the COVID-19 pandemic, many guidelines have recommended the use of personal protective equipment, a rubber dam, and a high-velocity suction device to reduce the spread of aerosols during dental procedures.^1,7,8^ Recently, extraoral suction (EOS) has been proposed as an additional device that could help reduce the spread of aerosols and splatters in dental practice. However, the efficacy of commercially available EOS has never been thoroughly investigated. Therefore, the present study aimed to evaluate the effectiveness of the EOS device in reducing the spread of aerosols and splatters during ultrasonic scaling.

## Methods

The experiment was conducted in a dental operatory unit using a dental manikin head (KaVo Dental Technologies, Charlotte, North Carolina, USA). The dental chair was set in a reclined position, and the manikin was placed on the headrest of the chair as in the dental scaling procedure. The floor of the manikin’s oral cavity was set at 65 cm above the floor. The doors of the dental operatory room were closed, and the air-conditioning and exhaust air systems were turned off during the scaling treatment to avoid air currents that could interfere with the splatter and aerosol patterns. A magnetostrictive ultrasonic scaler (Superson Merk III, Thai Dental Products Co., Ltd., Bangkok, Thailand) was set to operate at 25,000 Hz with a full water coolant supply.

### Detection of bacterial aerosols

**Figure 1:**
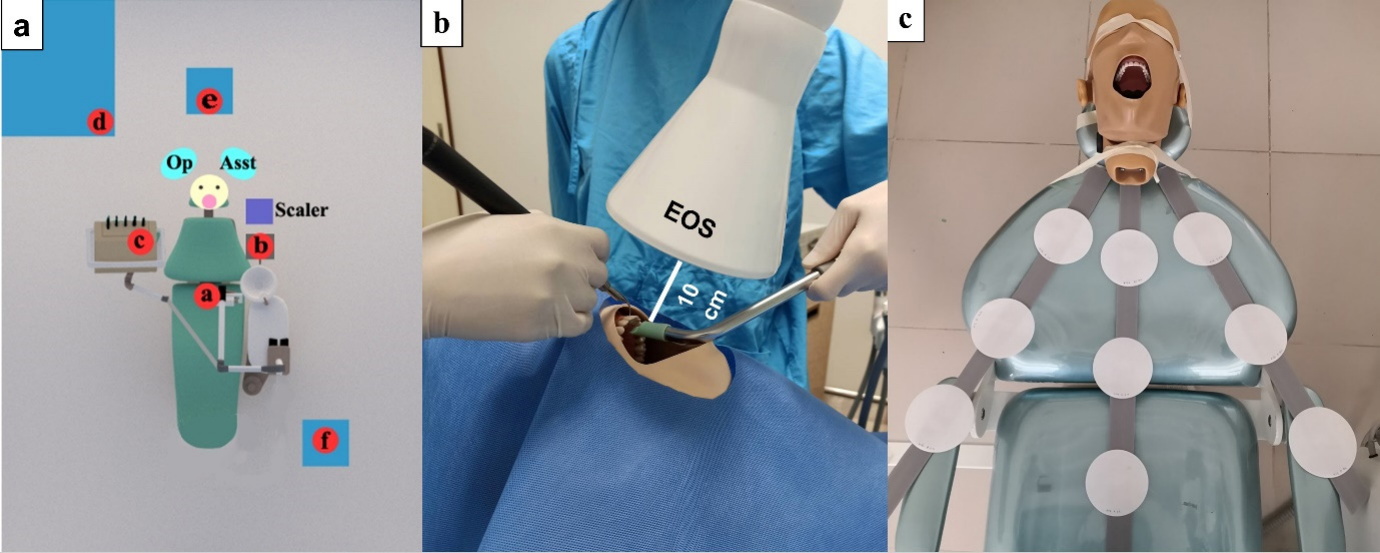
(a) Positions of the sampling sites in the dental operatory unit (Op, operator; Asst, assistant). (b) Orientation of extraoral suction head 10 cm from the working site and parallel to the mouth opening. (c) Placement of the filter paper discs.

To simulate the spreading characteristics of microorganisms in airborne particles generated during ultrasonic scaling, a suspension of *Lactobacillus acidophilus*recovered from anacidophilus probiotic (Nature’s Bounty, Inc., Bohemia, New York, USA) was cultured and added to sterile distilled water. The water containing 10^7^ CFU/mL of *L. acidophilus*was then used as a water coolant supply for an ultrasonic scaler. A settle plate sampler method, as described by Johnston et al,^9^ was used to detect the airborne bacteria contamination in the study. de Man–Rogosa–Sharpe (MRS) agar culture plates (Difco, Sparks, Maryland, USA) were placed at six selected sampling sites (a, b, c, d, e, and f), as shown in Figure 1a. The details of each sampling site are illustrated and described in Table 1. The culture medium plates were exposed to the air for 30 minutes before the scaling procedure to determine the background bacterial contamination in the room. After that, the new cultured plates were placed at the same selected sites, and the scaling was performed for 10 minutes with simultaneous use of (1) a high-volume evacuator (HVE) suction device alone (control) or (2) both HVE and EOS devices (HVE+EOS). The EOS device used in the study was the Elefas cavitation and aerosol system (CAS-350; Xianyang Raysun Foryou Medical Equipment Co., Ltd, Xianyang, Shaanxi, China), which produced a negative pressure of 20 kPa and contained high-efficiency particulate air (HEPA) filters. The EOS device has a suction rate of 4300 L/min and typically produces noise of approximately 50 decibels. The suction devices were operated throughout the scaling procedure, and the head of the EOS was oriented parallel to the opening of the oral cavity at 10 cm away from it, as shown in Figure 1 band Supplementary file 1. Throughout the scaling procedure, the culture plates were exposed to the air (10 minutes), and the plates were kept exposed for another 20 minutes after the scaling was completed. After each session, the air-conditioning and exhaust air systems were turned on for 1 hour, and a pair of 36-W ultraviolet C (UVC) germicidal light bulbs (G36T8; Philips, Amsterdam, Netherlands), which emit ultraviolet radiation of 253.7 nm, was operated for 30 minutes to decontaminate the room air. After collecting the samples, the bacterial culture plates were incubated anaerobically at 37±0.5°C for 48 hours. Then the bacterial colonies that had grown on the agar plates were counted, and the number of colonies was expressed as colony-forming units/plate (CFU/plate).

**Table 1:** Description of the sampling site and bacterial colony counts (CFU/plate) found at eachsampling site

**Site**	**Site description**	**Before scaling** **(n=5)** **Mean ± SD**	**HVE** **(n=5)** **Mean ± SD**	**HVE+EOS** **(n=5)** **Mean ± SD**	***P *** value*
a	50 cm horizontally from the oral cavity and 60 cm above the floor, located around the middle of the chair	0	23.40±11.37	6.00±2.83	0.025
b	60 cm horizontally from the oral cavity and 75 cm above the floor, located near the dental unit spittoon	0.50±0.57	19.20±9.07	18.80±8.07	0.943
c	60 cm horizontally from the oral cavity and 85 cm above the floor, located on the treatment tray	0.25±0.5	20.20±8.76	7.80±3.56	0.019
d	120 cm horizontally from the oral cavity and 75 cm above the floor, located on the bench	0.25±0.5	13.40±5.03	12.80±4.44	0.846
e	60 cm horizontally from the oral cavity and 70 cm above the floor, located behind the operator	0.5±0.57	19.20±3.90	5.60±3.58	< 0.001
f	150 cm horizontally from the oral cavity and 180 cm above the floor, located on the left side of the patient	1.5±1.29	21.40±11.44	5.80±3.63	< 0.001

*Independent sample t-test, the statistical analysis compared between HVE and HVE+EOS groups, significant at the P < 0.05 level. EOS, extra-oral suction; HVE, high-volume evacuator.

### Detection of splatter pattern 

Grade 1 white cellulose filter paper discs (11-cm diameter) (Double Ring, Hangzhou Ocome Technology Co. Ltd., Hangzhou, Zhejiang, China) were used in the study. The paper discs were set at three directions from the center of the mouth, corresponding to the 4, 6, and 8 o’clock positions, and the discs were placed at a distance of 20, 40, and 60 cm from the center of the mouth, as shown in Figure 1c. The paper discs were also placed on the wrists, chest, abdomen, and lap of the operator and assistant.

Fluorescein dye (Sigma-Aldrich, St. Louis, Missouri, USA) was used as the water supply for the ultrasonic scaler at a concentration of 0.1% dye solution. Full-mouth scaling was performed on a phantom jaw for 5 minutes using the ultrasonic scaler together and, simultaneously, with (1) HVE (control) or (2) HVE+EOS. After the scaling was completed, the fluorescent stain on the filter paper was visualized and imaged under ultraviolet light in complete darkness. Then the images were analyzed using ImageJ (NIH free software, Bethesda, Maryland, USA) to analyze the area of the fluorescent stain on filter papers.

### Statistical analysis

All the experiments were repeated five times. Statistical analysis was performed using SPSS 18 (IBM, Armonk, New York, USA). Differences between the two groups were analyzed using the independent sample t-test or Mann-Whitney U test. Differences for more than two groups were compared using two-way ANOVA followed by the multiple comparisons test; statistical significance was set at *P* < 0.05.

## Results

### Dissemination of bacterial aerosols

**Figure 2:**
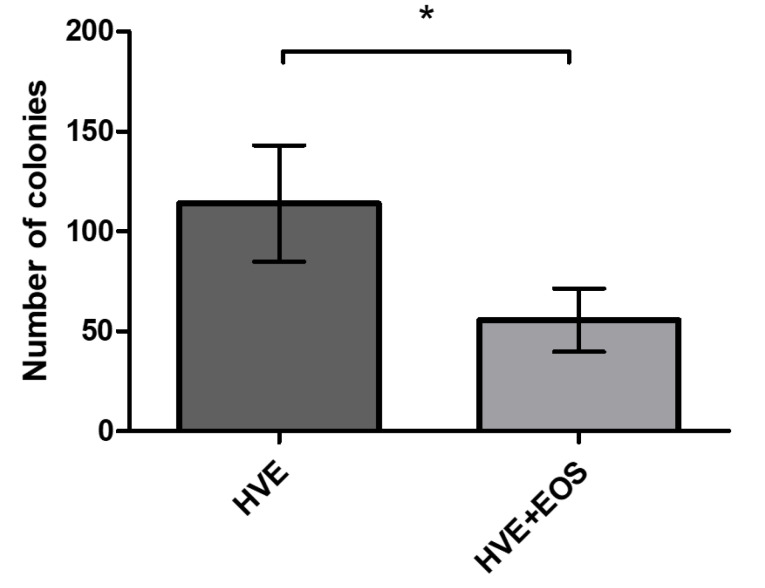
The total number of bacterial colonies counted in each group. EOS, extraoral suction; HVE, high-volume evacuator. *Indicate significant difference (*P* < 0.001).

Table 1 presents bacterial colony counts on the culture medium plates. Compared with the background bacterial aerosols, the scaling procedure with either the HVE or HVE+EOS increased bacterial contamination in the room. The airborne bacteria could travel as far as 150 cm horizontally and 115 cm vertically from the oral cavity. The area with the maximum contamination was at a distance of 50 cm horizontally from the oral cavity located at the middle of the dental chair. The EOS significantly reduced the number of bacterial colonies in most of the sampling sites. According to the total number of bacterial colonies found at each sampling site, the HVE+EOS significantly reduced the bacterial contamination in the room air (Figure 2).

### Dissemination of splatter pattern

The fluorescein stain on the filter paper was analyzed, and the percentage of the surface area of the stain detected on the filter paper discs was recorded. The dissemination pattern and the distance of the stained area from the oral cavity are shown in Table 2. The splatter pattern of the stain was in the range of a 20-cm radius from the oral cavity. The maximum contaminated area was found at the 4 o’clock position in both the HVE and HVE+EOS groups; the 8 o’clock position was the least contaminated area. The combination of the HVE and EOS significantly decreased the contaminated surface area compared with the HVE alone. The contamination was found on the wrists, chest, abdomen, and lap of the operator and assistant (Tables 3 and 4). The maximum contaminated surface area detected on the assistant was on the lap. In contrast, both the lap and right wrist were the most contaminated surface areas on the operator. The use of the EOS significantly reduced the surface contamination on the operator’s abdomen and wrist and the assistant’s lap.

**Table 2:** The surface area of contamination regarding the positions and distances from the oral cavity

	**Groups**					
	HVE (% surface area)* **(n=5)** **Mean ± S**D			HVE+EOS (% surface area)* **(n=5)** **Mean ± SD**		
Positions/ Distances	4 O’clock	6 O’clock	8 O’clock	4 O’clock	6 O’clock	8 O’clock
20 cm	1.237±0.25^1^A	0.413±0.183^B^	0.343±0.238^BC^	0.842±0.102^CD^	0.163±0.079^C^	0.103±0.066^EC^
40 cm	0	0	0	0	0	0
60 cm	0	0	0	0	0	0

*Two-way ANOVA with multiple comparisons test, significant at the *P* < 0.05 level, the same letter in the superscript is not significantly different from another. EOS, extra-oral suction; HVE, high-volume evacuator.

**Table 3:** Area of contamination on the operator

**Area of operator**	**Groups**		P value*
	**HVE (n=5)** **(% surface area)** **Mean ± SD**	**HVE+EOS (n=5)** **(% surface area)** **Mean ± SD**	
Right wrist	6.268±0.729	3.726±1.649	0.022
Left wrist	0.346±0.170	0.0736±0.0293	0.022
Chest	0.315±0.275	0.150±0.092	0.237
Abdomen	0.652±0.241	0.115±0.068	0.006
Lap	10.076±5.483	3.017±0.680	0.081

*Independent sample t-test, significant at the *P* < 0.05 level. EOS, extra-oral suction, HVE, high-volume evacuator.

**Table 4:** Area of contamination on the assistant

**Area of assistant**	**Group**		***P *** value*
	**HVE (n=5)** **(% surface area)** **Mean ± SD**	**HVE+EOS (n=5)** **(% surface area)** **Mean ± SD**	
Right wrist	0.047±0.040	0.015±0.032	0.056
Left wrist	0.058±0.090	0.052±0.019	0.690
Chest	0.068±0.009	0.005±0.012	0.841
Abdomen	0.027±0.051	0.014±0.020	1.00
Lap	1.606±1.674	0.130±0.109	0.008

*Independent sample t-test or Mann-Whitney U test, significant at the *P* < 0.05 level. EOS, extra-oral suction; HVE, high-volume evacuator.

## Discussion

Dental procedures generate airborne particles and droplets contaminated with microorganisms that could cause the spread of infectious diseases. Ultrasonic scaling, a general dental procedure usually performed daily, produces the most aerosols and splatters that could lead to an increased risk of disease transmission and infection in the dental office.^1^ Many guidelines recommend using high-velocity suction, HEPA room air filters, and ultraviolet treatment of the ventilation system to reduce bioaerosol contamination.^1,7,8^ However, some of the recommendations might not be practical in some dental settings. EOS has been suggested as an additional device that could reduce the spread of splatters and bioaerosols in the dental clinic, but evidence for its effectiveness is insufficient. Therefore, the present study investigated the efficacy of EOS for aerosol and splatter reduction during dental treatments. The present study used the scaling procedure as a representative dental treatment because ultrasonic scaling has been demonstrated to produce the most aerosol and splatter contamination, suggesting a high risk for disease transmission in the dental clinic.

*Lactobacillus*spp. are rod-shaped, gram-positive, lactic acid-producing bacteria commonly found in the oral cavity.^10,11^ In this study, *L. acidophilus*,one of the *Lactobacillus*spp. that can be detected in the oral cavity and is considered a probiotic bacteria in the oral and gastrointestinal tracts,^10,12,13^ was used to represent the spread of bacterial air contamination during ultrasonic scaling. The study also used MRS agar, a selective medium for cultivating *Lactobacillus* spp.,^14^ to confirm that the detected bacterial aerosols were generated from the scaling procedure.

The results showed that ultrasonic scaling generated bacterial aerosols that could be disseminated throughout the dental operatory and that bacterial aerosols could travel as far as 150 cm horizontally from the oral cavity, increasing the risk of disease transmission in dental practice. Our finding was consistent with a study by Chuang et al,^15^ who reported the spread of airborne bacteria in various distances and directions during scaling treatments on periodontitis patients. Furthermore, the maximum air contamination was observed at a horizontal distance of 50 cm from the oral cavity; the sampling site was located around the middle of the dental chair (Figure 1a; site a). This was consistent with a previous study, which found the highest bacterial contamination at the patient’s abdominal area during ultrasonic scaling.^6^

The combination of EOS and HVE significantly decreased bacterial air contamination at most sampling sites (Table 1). No significant differences in the number of bacterial colonies were observed at sampling sites b and d (Figure 1) because the EOS head was placed 10 cm away from the oral cavity in the present study. In contrast, Motegi et al^16^ reported the position of the EOS head at 5 cm from the treatment site. The greater distance of the EOS head from the aerosol-generating source might have decreased its full power. Nevertheless, the EOS significantly reduced the total number of bacteria contaminating the air in the room (Figure 2). The results of the present study are consistent with a study by Motegi et al,^16^ who found a reduction in the number of bacterial colonies with the use of EOS+HVE during subgingival scaling in a periodontitis patient. Teanpaisan et al^17^ also reported a decrease in bacterial CFU using a modified household wet-dry-blow vacuum cleaner during ultrasonic scaling.

Fluorescein sodium is a nontoxic yellow dye that produces an intense green fluorescence.^18^ The dye has commonly been used to detect ophthalmic and skin lesions.^18,19^ It has also been used as a water tracer.^20^ In this study, we added the fluorescein dye into the water supply of the ultrasonic scaler to trace the dissemination of water during the scaling procedure. The splatter pattern from the scaling procedure was detected within 20 cm of the oral cavity. The maximum and minimum contaminated surfaces were found at the 4 o’clock and 8 o’clock positions, respectively (Table 2), consistent with a previous study, which found that the 4 o’clock and 8 o’clock positions were the most and least contaminated areas, respectively, in a 30-cm radius.^21^ This suggested that secretions from the oral cavity could spread to 20‒30 cm away from the mouth and that the left-hand side of the patient was the riskiest area for disease transmission. The use of HVE+EOS significantly reduced the contaminated surface area compared with the use of HVE alone. The contamination was also detected on many parts of the body, especially on the lap, abdomen, and wrists of the operator and assistant, indicating a risk of disease transmission from patient to dental care professionals (Tables 3 and 4). Personal protective equipment, such as long-sleeved medical gowns, gloves, masks, face shields, and shoe covers, is important. The EOS reduced contaminated surface areas on every part of the assistant’s and operator’s bodies; however, significant differences were only observed on the abdomen of the operator and lap of the assistant.

Although the EOS device was shown to affect bioaerosol and splatter reduction, there were some limitations and drawbacks regarding its application. To achieve its full function, the EOS head must be placed close to the operating site, and this might interfere with the field of operation and make it harder to perform dental treatments. Moreover, long-term exposure to loud noise generated by the EOS device could cause impaired hearing in dental healthcare professionals.

## Conclusion

The EOS device effectively reduced the dissemination of the bioaerosols and splatters generated during ultrasonic scaling. The combination of HVE and EOS is an effective method in preventing the transmission of airborne particles and could be used as a new strategy for infection control and management in the dental clinic.

## Authors’ Contributions

Conceptualization: SH and WL. Methodology and experimental work: SH, WL, and YC. Analysis and interpretation of data: SH, WL, and YC. Drafting, reviewing, and editing: SH, WL, and RS. All authors have read and agreed to the published version of the manuscript.

## Acknowledgments

The authors thank the Dental Simulation Center, Faculty of Dentistry, Mahidol University, for the support on the instruments.

## Funding

None.

## Competing Interests

All authors have no conflict of interests to report.

## Ethics Approval

Not applicable.

## Supplementary Materials

Supplementary file 1. VDO1 illustrating the function of EOS during an ultrasonic scaling.
